# Circular RNA hsa_circ_0002360 promotes non-small cell lung cancer progression through upregulating matrix metalloproteinase 16 and sponging multiple micorRNAs

**DOI:** 10.1080/21655979.2021.1999370

**Published:** 2021-12-25

**Authors:** Yunting Zhang, Shaolin Zeng, Tao Wang

**Affiliations:** Department of Respiratory and Critical Care Medicine, Renmin Hospital of Wuhan University, Wuhan, Hubei, China

**Keywords:** Non-small cell lung cancer, circ_0002360, microRNAs, matrix metalloproteinase 16

## Abstract

Dysregulated circular RNAs (circRNAs) are involved in the progression of non-small cell lung cancer (NSCLC). However, the role of has_circ_0002360 (circ_0002360) in NSCLC has rarely been reported. In this study, circ_0002360 expression in NSCLC tissues and cell lines was measured using microarray data and quantitative real-time PCR (qRT-PCR). After gain-of-function and loss-of-function, cell models were established; 5-bromo-2-deoxyuridine (BrdU) and transwell assays were conducted to detect NSCLC cell growth, migration, and invasion. What is more, bioinformatic analysis and dual-luciferase reporter assay were adopted to show how circ_0002360, microRNAs (miR-127-5p, miR-145-5p, miR-585-3p, and miR-758-3p), and matrix metalloproteinase 16 (MMP16) 3ʹUTR interact with each other. Western blotting was executed to probe the regulatory effects of circ_0002360 and these miRNAs on MMP16 protein expression in NSCLC cells. We found that circ_0002360 expression was raised in NSCLC tissues. High circ_0002360 expression predicted a short overall survival time for NSCLC patients. Circ_0002360 overexpression promoted NSCLC cell proliferative, migrative, and invasive abilities, and circ_0002360 depletion worked oppositely. MiR-127-5p, miR-145-5p, miR-585-3p, and miR-758-3p were the targets of circ_0002360, and circ_0002360 could regulate MMP16 expression by competitively binding with the above miRNAs. In summary, circ_0002360 serves as a competitive endogenous RNA to raise MMP16 expressions by competitively binding to miR-127-5p, miR-145-5p, miR-585-3p, and miR-758-3p, thereby promoting NSCLC progression.

## Graphical Abstract

**Figure:**
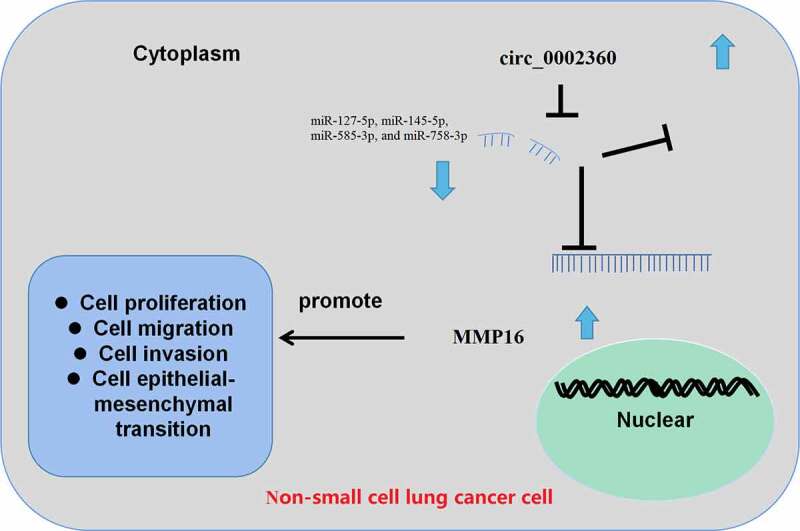

## Introduction

Lung cancer (LC) is the malignancy with the highest morbidity and mortality in the globe [1], and non-small cell lung cancer (NSCLC), making up for around 85% of all LC cases [2]. Important advances have been made in treating NSCLC; however, the prognosis of NSCLC sufferers is still not satisfactory, with a five-year overall survival rate only around 15% [1–4]. It is vital to study the molecular mechanisms associated with NSCLC progression for improving the diagnosis and treatment of NSCLC [4].

Circular RNAs (circRNAs) are non-coding RNAs (ncRNAs) with covalently closed-loop structures formed by linear mRNA back-splicing [5]. CircRNAs are characterized by high abundance, high stability, and high conservation, so circRNAs have advantages as diagnostic markers for diseases [6]. CircRNAs are closely associated with the tumorigenesis of cancer [7,8]. For instance, circ_0002483 is lowly expressed in NSCLC tissues and cell lines, and its overexpression greatly restrains the proliferation and migration of NSCLC cells [9]. In recent years, it has been a hotspot in cancer research that circRNAs competitively bind to microRNA (miRNA) to modulate gene expression [10]. For instance, circ-ITGA7 may function as a competing endogenous RNA (ceRNA) to upregulate ASXL1 expressions through adsorbing miR-3187-3p, thus suppressing colorectal cancer proliferation [11]. Interestingly, a recent study reports that hsa_circ_0002360 was highly expressed in lung adenocarcinoma [12]. In the present study, a bioinformatics analysis suggested that circ_0002360 was highly expressed in NSCLC. However, the biological function and related ceRNA mechanism of circ_0002360 in NSCLC progression are unclear.

The scientific hypothesis of the present work is that circ_0002360 may be a regulator of NSCLC progression. Our study can improve the understanding of the mechanism of NSCLC progression and provide novel candidate targets for NSCLC diagnosis and treatment. Here, we report that circ_0002360 expression is elevated in NSCLC, and circ_0002360 increases matrix metalloproteinase 16 (MMP16) expression by competitively binding to miR-127-5p, miR-145-5p, miR-585-3p, and miR-758-3p, thereby promoting the progression of NSCLC.

## Materials and methods

### Patient tissue specimens

We collected 32 pairs of NSCLC tissues and adjacent tissues from the Renmin Hospital of Wuhan University. All tissue specimens were stored in liquid nitrogen. All patients provided the written informed consent, and the collection of human samples was approved by the Ethics Committee of Renmin Hospital of Wuhan University.

### Bioinformatics analysis

Datasets GSE112214 and GSE101586 were downloaded from Gene Expression Omnibus (GEO). GSE112214 contains microarray expression profiling data of circRNAs of three NSCLC samples and three matched adjacent normal samples. GSE101586 contains microarray expression profiling data of circRNAs of five NSCLC samples and five matched adjacent normal samples. The differentially expressed circRNAs were screened with an online analysis tool, GEO2R. *P* < 0.05 and │log_2_ (Fold Change)│>2 were used as the cutoff criterion for screening the differentially expressed circRNAs. The binding sequences between circ_0002360 and the miRNAs were analyzed by CircInteractome (https://circinteractome.nia.nih.gov/) [13]. The binding sequences between the miRNAs and MMP16 3ʹUTR were predicted by TargetScan (http://www.targetscan.org/vert_71/) [14]. To perform gene set enrichment analysis (GSEA), the R package of the algorithm was downloaded from the homepage (http://www.gsea-msigdb.org/gsea) [15], and the data of NSCLC tissues of TCGA database (http://cancergenome.nih.gov/) [16] were downloaded and analyzed by the algorithm.

### Cell culture and transfection

Human NSCLC cell lines (A549, HCC827, NCI-H23, and H125) and normal lung epithelial cell line (BEAS-2B) were obtained from Shanghai Institute of Cell Biology, Chinese Academy of Sciences (Shanghai, China). These cells were cultured in RPMI-1640 medium (Gibco, Carlsbad, CA, USA) with 10% fetal bovine serum (Gibco), 100 U/mL penicillin, and 100 μg/mL streptomycin (Invitrogen, Carlsbad, CA, USA) in 5% CO_2_ at 37°C. pcDNA-circ_0002360 (circ-OE), empty vector (NC), circ_ 0002360 siRNA (si-circ#1: 5ʹ-GTCAGATGCAGGGGAAAAGCT-3ʹ and si-circ#2: 5ʹ-AGAGTCAGATGCAGGGGAAAA-3ʹ), scramble siRNA (si-NC: 5ʹ-UUCUCCGAACGUGUCACGUTT-3ʹ), miRNA mimics (miR-127-5p: 5ʹ-CUGAAGCUCAGAGGGCUCUGAU-3ʹ; miR-145-5p:5ʹ-GUCCAGUUUUCCCAGGAAUCCCU-3ʹ; miR-585-3p: 5ʹ-UGGGCCGUAUCUGUAUGCUA-3ʹ; miR-758-3p: 5ʹ-UUUGUGACCUGGUCCACUAACC-3ʹ), and negative control (mimics NC: 5ʹ-UUCUCCGAACGUGUCACGUTT-3ʹ) were purchased from Invitrogen. The transfection was conducted with Lipofectamine® 2000 (Invitrogen). Briefly, HCC827 and A549 cells were cultured in a serum-free medium for 12 h. The transfection reagent and oligonucleotide/plasmids were diluted in a serum-free medium, respectively, and then mixed and incubated for 1 h at room temperature. Next, the transfection reagent was added to transfect the cells for 12 h. 12 h later, the medium was replaced by complete medium, and the cell culture was continued for another 24 h. Subsequently, the cells were harvested, and quantitative real-time PCR (qRT-PCR) was performed to detect the transfection efficiency.

### qRT-PCR

The total RNA was extracted from NSCLC tissues and transfected HCC827 and A549 cells with a TRIzol kit (Yeasen Biotech, Shanghai, China). cDNA synthesis was conducted with a TaqMan MicroRNA reverse transcription kit (Applied Biosystems, Foster City, CA, USA) for miRNAs and with a PrimeScript RT Master Mix Kit (TaKaRa, Dalian, China) for circ_0002360 and MMP16, respectively. Next, with cDNA as the template, qRT-PCR was conducted to examine the relative expressions of circ_0002360 and MMP16 by the SYBR® Premix Ex Taq^TM^ II kit (TaKaRa). Besides, a stem-loop primer SYBR Green qRT-PCR kit (Synbio Tech, Suzhou, China) was used for qRT-PCR to measure miRNAs’ expression levels. With U6 and GAPDH as internal references, the relative expressions of circ_0002360, miRNAs, and MMP16 were calculated by 2^−ΔΔCt^. Primer sequences are shown in Table 1. To identify the subcellular localization of circ_0002360, circ_A PARIS™ kit (Thermo Fisher Scientific, Waltham, MA, USA) was used for subcellular fractionation of HCC827 and A549 cells. Then, circ_0002360 expression in the cytoplasm and nucleus of the cells was examined by qRT-PCR, with GAPDH and U6 as the cytoplasmic control and nuclear control, respectively.

**Table 1.** Primer sequences

Name	Primer sequences	
Circ_0002360		Forward: 5′-CCACTCCACTGCCTTTAACC-3′
		Reverse: 5′-TGATTTTGATGGCTCTGTGG-3′
MiR-127-5p		Forward: 5ʹ-CTCTTCAAGCTCCAAACCAAAC-3ʹ
		Reverse: 5′-GTATCCACCAGAACCACCAGG-3′
MiR-145-5p		Forward: 5ʹ-GTCCAGTTTTCCCAGGAATC-3ʹ
		Reverse: 5ʹ-AGAACAGTATTTCCAGGAAT-3ʹ
MiR-585-3p		Forward: 5′-TCGGCAGGTGGGCGTATCTGT-3′
		Reverse: 5′-CTCAACTGGTGTCGTGGA-3′
MiR-758-3p		Forward: 5′-CTCCAGCTGGGTTTGTGACCTGGTCCA-3′
		Reverse: 5′-CTCAACTGGTGTCGTGGAGTCGGCAATTCAGTTG AGGGTTAGTG-3′
MMP16		Forward: 5′-CTATTCTTCGTCGTGAGATGT-3′
		Reverse: 5′-CCGTCGCTATTTTCATAAAC-3′
U6		Forward: 5ʹ-GCTTCGGCAGCACATATACTAAAAT-3ʹ
		Reverse: 5ʹ-CGCTTCACGAATTTGCGTGTCAT-3ʹ
GAPDH		Forward: 5ʹ-AATTCCATGGCACCGTCAAG-3ʹ
		Reverse: 5ʹ-TGGACTCCACGACGTACTC-3ʹ

### BrdU assay

Transfected HCC827 and A549 cells (1 × 10^5^ cells/ml) were seeded in 35 mm diameter dishes (containing a slide inside) and cultured for 1 day and synchronized with a medium containing 0.4% FBS for 3 days. Subsequently, 1.0 mg/ml BrdU reagent (BD Pharmingen, San Diego, CA, USA) was loaded and subsequently incubated at 37°C for 4 h. Next, the medium was discarded, and the slides were washed three times with phosphate buffer saline (PBS) and subsequently fixed with methanol for 10 min, then blocked with 5% normal rabbit serum, and DNA was denatured by formalin. Next, the cells were rinsed in PBS and then incubated with an anti-BrdU antibody (1:500, Beyotime, Shanghai, China). After incubation for 2 h at ambient temperature, the cells were incubated with secondary antibody (1:500, Beyotime) for 1 h at room temperature and then rinsed three times in PBS. After that, the cells were stained with DAPI staining solution (Beyotime, Haimen, China). Finally, the total numbers of cells and BrdU-positive cells in random five high-power visual fields were counted under a fluorescence microscope.

### Transwell assay

The transwell inserts (Corning Life Sciences, Corning, NY, USA) were adopted to examine the migrative and invasive abilities of HCC827 and A549 cells. As for migration assays, the cells suspended in serum-free medium were inoculated into the upper compartment of Transwell inserts (1 × 10^4^ cells per well), and 500 μL of medium with 10% FBS was loaded into the lower chamber and subsequently the cells were cultured in 5% CO_2_ at 37°C for 24 h. Next, the non-migrated cells were scraped off with a cotton wipe. Then, the inserts were carefully rinsed with PBS twice, and the cells on the lower surface of the transwell membrane were subsequently fixed with 95% methanol, and stained with 0.5% crystal violet solution for 15 min. Finally, the number of cells in five randomly selected visual fields of the membrane was counted. For invasion assay, the membranes of the inserts were coated with a layer of Matrigel, and the remaining steps followed the migration assay.

### Western blot assay

Transfected HCC827 and A549 cells were lysed in radioimmunoprecipitation assay (RIPA) lysis buffer (Beyotime, Shanghai, China), and the protein concentration was subsequently examined by a bicinchoninic acid protein assay kit (Beyotime). The proteins were subsequently dissolved by sodium dodecyl sulfate-polyacrylamide gel electrophoresis and transferred to the polyvinylidene fluoride (PVDF) membrane, which were subsequently blocked with 5% skimmed milk for 1 h at ambient temperature and then incubated with anti-MMP16 antibody (1:1000, ab73877) and β-actin antibody (1:2000, ab8226) overnight at 4°C. On next day, the membranes were washed in Tris-buffered saline Tween (TBST) and then incubated with horseradish peroxidase-conjugated secondary antibody (1:1000, ab205718 or ab205719) for 50 min at room temperature. All antibodies were purchased from Abcam (Cambridge, UK). Next, the protein bands were developed by an Amersham Imager 600 (GEHealthcare, Chicago, IL, USA) with an electrochemiluminescence kit (Biosharp, Hefei, China).

### Dual-luciferase reporter gene assay

The binding fragments of circ_0002360 and MMP16 3ʹUTR with miRNAs were amplified by PCR. The amplified products were subsequently inserted into the pGL3-Promoter plasmid vector (Promega, Madison, WI, USA) to construct circ_0002360 and MMP16 wild-type (wt) reporters (circ_0002360-wt, and MMP16-wt). Also, circ_0002360 and MMP16 mutant (mut) reporters (circ_0002360-mut and MMP16-mut) were constructed by a QuickChange Multiple Site-directed Mutagenesis Kit (Stratagene, La Jolla, CA, USA). Additionally, the recombinant plasmids were co-transfected into HCC827 and A549 cells with four miRNAs (miR-127-5p, miR-145-5p, miR-585-3p, and miR-758-3p) mimics and mimics NC. Forty-eight hours later, the luciferase activity of the cells in each group was examined by the Dual-Luciferase Reporter Assay System (Promega, Madison, WI, USA) as required by the procedures.

### Statistical analysis

All data were expressed as ‘mean ± SD’, and statistical analyses were conducted using GraphPad Prism 8 (GraphPad Software, Inc., La Jolla, CA, USA) and SPSS version 20.0 (SPSS Inc., Chicago, IL, USA). Independent sample *t*-test and one-way analysis of variance were executed for the comparisons between two groups and among multiple groups, respectively. Survival analysis was conducted with a log-rank test. Gene expression correlations were analyzed by Pearson’s correlation coefficient analysis. Statistically, *P* < 0.05 is significant.

## Results

In this work, using bioinformatics analysis and *in vitro* experiments, we found that circ_0002360 was highly expressed in NSCLC tissues, and it promoted the malignancy of NSCLC cells. Mechanistically, circ_0002360 could induce the expression of MMP16 via repressing miR-127-5p, miR-145-5p, miR-585-3p, and miR-758-3p.

### Circ_0002360 expression is up-regulated in NSCLC

**Figure 1.**
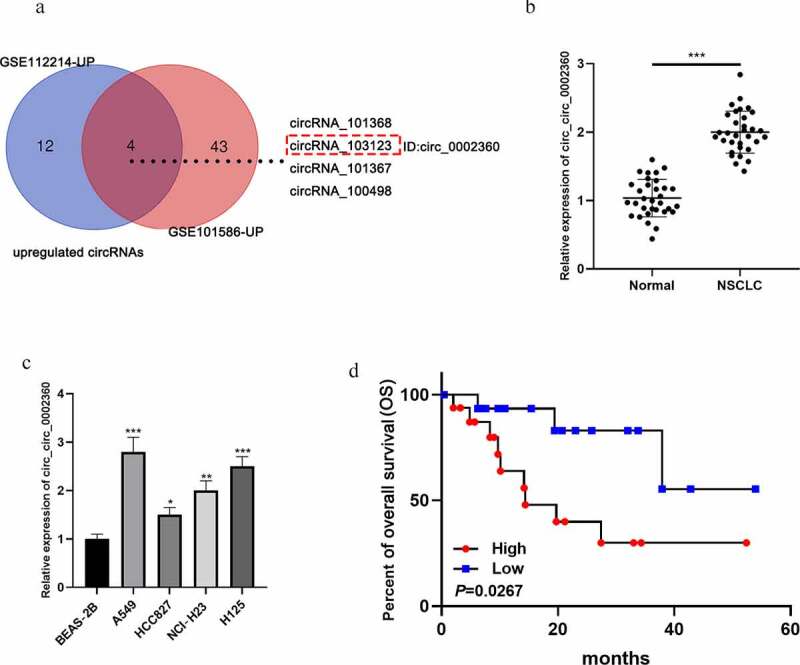
*Circ_0002360 is up-regulated in NSCLC* A. The significantly upregulated circRNAs in GSE112214 and GSE101586. B. The expression of circ_0002360 in NSCLC tissues and adjacent tissues was detected by qRT-PCR. C. The expression of circ_0002360 in NSCLC cell lines (A549, HCC827, NCI-H23, and H125) and normal lung epithelial cell line (BEAS-2B) was detected by qRT-PCR. D. Kaplan–Meier curve was used to analyze the relationship between the expression of circ_0002360 and the overall survival time of NSCLC patients. *P < 0.05, **P < 0.01, and ***P < 0.001

First of all, circRNA expression profiles (GSE112214 and GSE101586) were analyzed, and four circRNAs (circRNA_101368, circRNA_103123, circRNA_101367, and circRNA_100498) were significantly up-regulated in the NSCLC tissues of both datasets (Figure 1a). Then, we further analyzed the expression characteristics of circRNA_103123 (circ_0002360) in NSCLC samples with qRT-PCR. It showed that the circ_0002360 expression level was remarkably higher in NSCLC tissues than that in adjacent tissues (Figure 1b). Additionally, circ_0002360 expression level was markedly raised in NSCLC cell lines (A549, HCC827, NCI-H23, and H125) as against that of normal lung epithelial cell line (BEAS-2B) (Figure 1c). Additionally, high expression of circ_0002360 was associated with the differentiation status of the tumor tissues (Table 2). Notably, the Kaplan–Meier curve showed that the up-regulation of circ_0002360 was associated with a shorter overall survival time of NSCLC sufferers (Figure 1d).

**Table 2.** Correlation between 0002360 expression and clinicopathological characteristics of the 32 NSCLC patients

Characteristics	Number	circ_0002360 expression		P value
		Low	High	
Age(years)				
≤ 50	9	5	4	1.000
> 50	23	11	12	
Gender				
Male	24	12	12	1.000
Female	8	4	4	
TMN stage				
I/II	19	11	8	0.472
III/IV	13	5	8	
Differentiation status				
Well/Moderate	13	11	2	0.004*
Poor	19	5	14	
Histological subtype				
Squamous carcinoma	14	8	6	0.722
Adenocarcinoma	18	8	10	

*P* value was calculated by Fisher’s exact test. * denotes *P* values less than 0.05.

### Circ_0002360 promotes NSCLC cell multiplication, migration, and invasion

**Figure 2.**
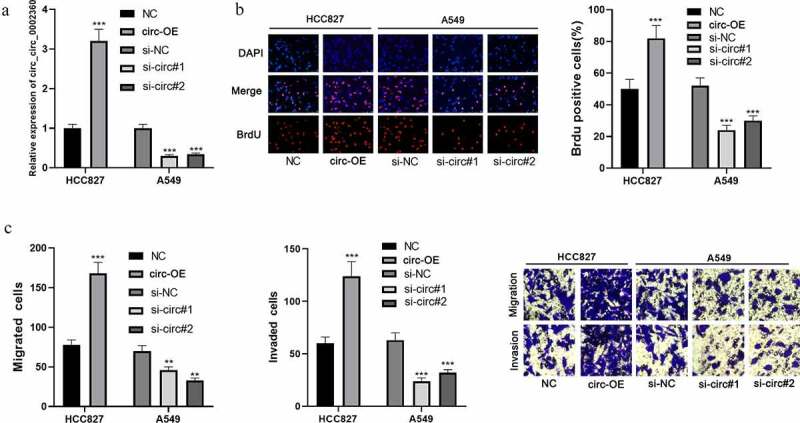
*Circ_0002360 promotes NSCLC cell proliferation, migration, and invasion* A. Circ_0002360 was overexpressed and depleted in HCC827 and A549 cell lines, respectively, and the expression of circ_0002360 was measured by qRT-PCR. B. BrdU assay was used to detect NSCLC cells’ proliferative ability. C. Transwell assay was used to detect the migration and invasion of NSCLC cells. NC, negative control plasmids; Circ-OE, circ_0002360 overexpression plasmids; si-NC, negative control siRNA; si-Circ, circ_0002360 siRNA. **P < 0.01, and ***P < 0.001

Next, pcDNA-circ_0002360 and si-circ_0002360 (si-circ#1 and si-circ#2) were transfected into HCC827 and A549 cells, respectively, to obtain circ_0002360 overexpression or knockdown cell model (Figure 2a). The BrdU assay showed that circ_0002360 overexpression markedly promoted HCC827 cell growth compared with the control group (Figure 2b). In addition, the transwell assay showed that circ_0002360 overexpression promoted HCC827 cell migration and invasion (Figure 2c). Circ_0002360 depletion remarkably inhibited A549 cell proliferation, migration, and invasion (Figure 2b and c). These experiments indicated that circ_0002360 promoted the malignancy of NSCLC.

### Circ_0002360 functions as a ceRNA and sponges multiple miRNAs

**Figure 3.**
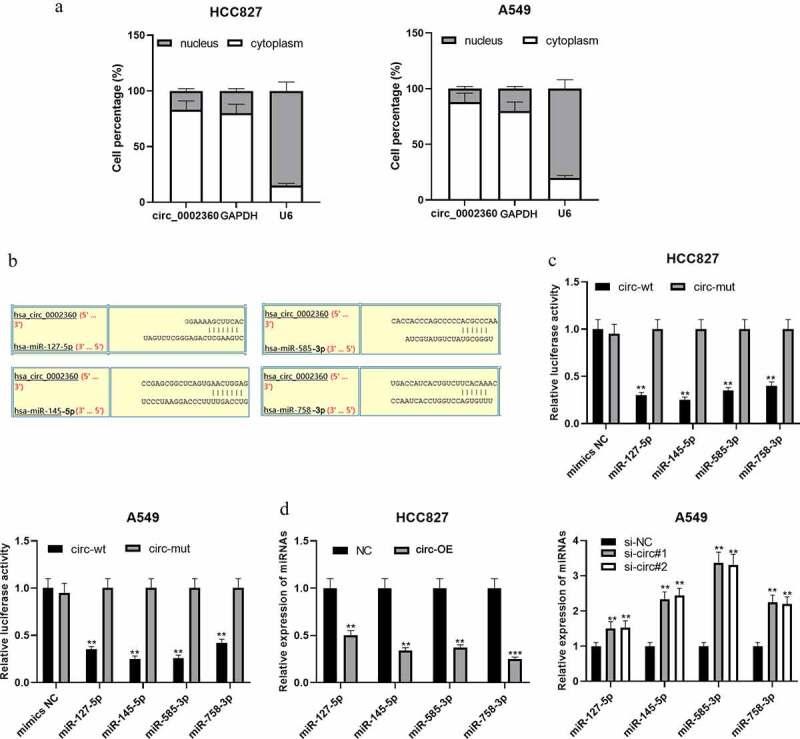
*Circ_0002360 functions as a ceRNA and sponges multiple miRNAs* A. The distribution of circ_0002360 in the cytoplasm and nucleus of NSCLC cell was detected by qRT-PCR. B. Bioinformatics was used to predict the potential targets of circ_0002360 and specific binding sites between circ_0002360 and miRNAs. C. Dual-luciferase reporter gene assay was used to validate the targeting relationships between circ_0002360 and miR-127-5p/miR-145-5p/miR-585-3p/miR-758-3p. D. qRT-PCR was conducted to analyze the effects of circ_0002360 overexpression or knockdown on the expression of miR-127-5p, miR-145-5p, miR-585-3p, and miR-758-3p. NC, negative control plasmids; Circ-OE, circ_0002360 overexpression plasmids; si-NC, negative control siRNA; si-Circ, circ_0002360 siRNA; circ-wt, wide-type circ_0002360 reporter; circ-mut, mutant-type circ_0002360 reporter. **P < 0.01, and ***P < 0.001

CircRNAs can act as ceRNAs and competitively bind with miRNAs [17]. qRT-PCR showed that circ_0002360 was mainly in the cytoplasm of NSCLC cells, suggesting that it could probably function as a ceRNA (Figure 3a). CircInteractome database showed that miR-127-5p, miR-145-5p, miR-585-3p, and miR-758-3p were the potential targets of circ_0002360 (Figure 3b). Dual-luciferase reporter gene assays highlighted that the up-regulation of these miRNAs repressed the activity of circ_0002360-wt in NSCLC cells, but that of circ_0002360-mut was not significantly affected (Figure 3c). qRT-PCR proved that circ_0002360 overexpression in HCC827 cell line resulted in a significant decrease in miR-127-5p, miR-145-5p, miR-585-3p, and miR-758-3p expression, while circ_0002360 knockdown worked oppositely in A549 cells (Figure 3d). The above results indicated that the above miRNAs were the targets of circ_0002360.

### MiR-127-5p, miR-145-5p, miR-585-3p, and miR-758-3p specifically regulate MMP16

**Figure 4.**
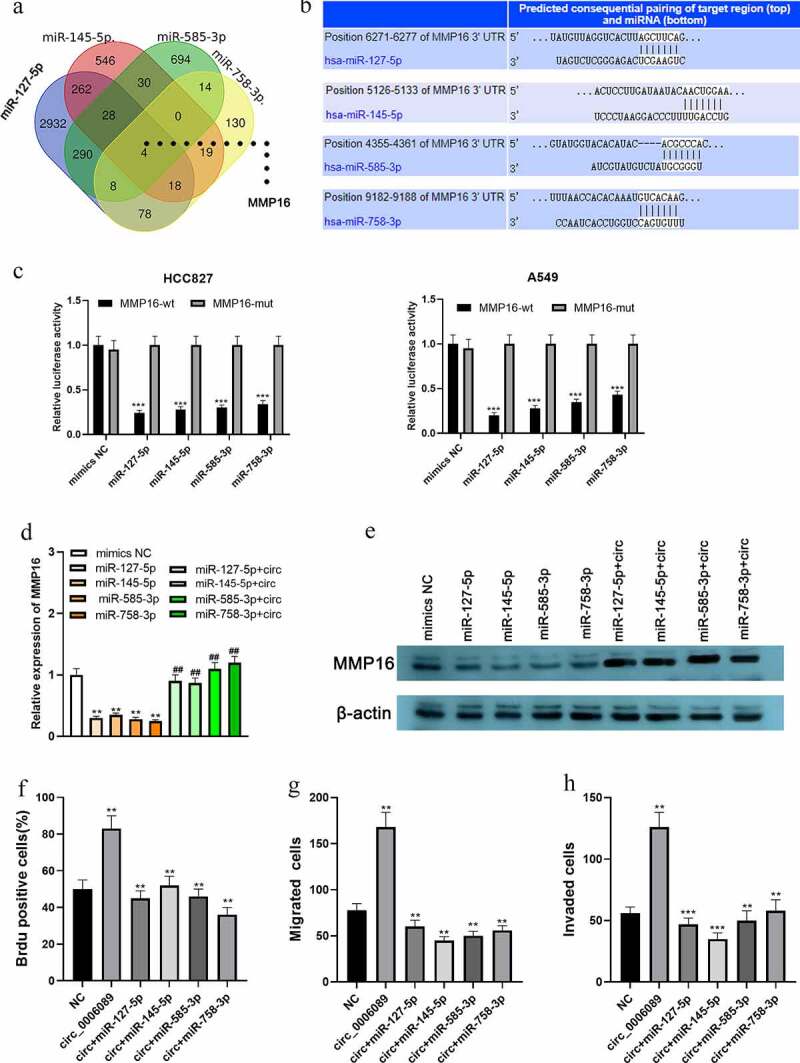
*miR-127-5p, miR-145-5p, miR-585-3p, and miR-758-3p specifically regulate MMP16* A. Venn diagram was used to present the common targets of miR-127-5p, miR-145-5p, miR-585-3p, and miR-758-3p. B. The binding sites between miR-127-5p/miR-145-5p/miR-585-3p/miR-758-3p and MMP16 3′-UTR were predicted by TargetScan database. C. Dual-luciferase reporter gene assay was adopted to validate the targeting relationship between MMP16 3′-UTR and the miRNAs. D&E. qRT-PCR and western blot assay were used to detect the expression of MMP16 in A549 cells after circ_0002360 and these miRNAs were overexpressed. F-H. BrdU assay and transwell assay were used to detect the proliferation, migration and invasion of NSCLC cells co-transfected with circ_0002360 overexpression vector and miRNA mimics, respectively. MMP16-wt, wide type MMP16 reporter; MMP16-mut, mutant type MMP16 reporter; mimic NC, negative control mimics; circ, circ_0002360 overexpression plasmids. *P < 0.05, **P < 0.01, and ***P < 0.001 vs control group. ^##^*P* < 0.01 vs miRNAs mimic group

**Figure 5.**
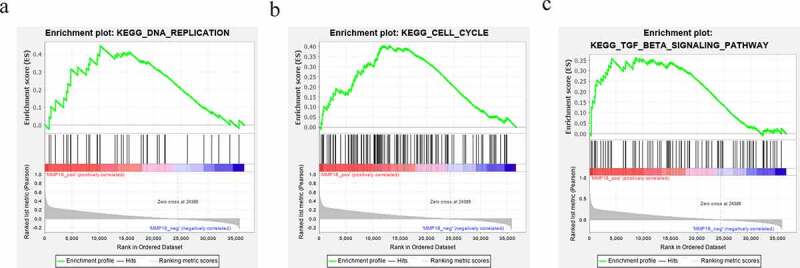
*MMP16 is associated with multiple cancer-related biological processes and signaling pathways*. A–C. GSEA indicated that MMP16 was associated with the activation of DNA replication (a), cell cycle (b), and TGF-β pathway (c)

Interestingly, bioinformatic analysis implied that there were base complementary binding sites between MMP16 3′-UTR and miR-127-5p, miR-145-5p, miR-585-3p, or miR-758-3p (Figure 4a and b). Consistently, the dual-luciferase reporter gene assay confirmed that MMP16 was a common target of miR-127-5p, miR-145-5p, miR-585-3p, and miR-758-3p (Figure 4c). Additionally, qRT-PCR and western blot showed that, in A549 cells, up-regulation of the aforementioned miRNAs markedly inhibited MMP16 mRNA and protein expressions as against the mimics-NC group, while circ_0002360 overexpression reversed this inhibitory effect (Figure 4d and e). The above results indicated that circ_0002360 increased MMP16 expression by targeting miR-127-5p, miR-145-5p, miR-585-3p, and miR-758-3p. In addition, the impacts of circ_0002360 overexpression on promoting NSCLC cell growth, migration, and invasion can be counteracted by upregulation of miR-127-5p, miR-145-5p, miR-585-3p, or miR-758-3p (figure 4f–h). Additionally, to show the biological function of MMP16 in NSCLC progression, GSEA was performed based on RNA sequencing data from the TCGA database, and the findings suggested that high expression of MMP16 was associated with the activation of DNA replication, cell cycle progression, and TGF-β signaling (Figure 5a–c). These data suggested that circ_0002360 promoted the expression of MMP16 via repressing multiple miRNAs to exert oncogenic effects on NSCLC progression.

## Discussion

CircRNAs were previously thought to be the byproducts of aberrant splicing in the transcriptome [18]. In recent years, the following biological functions of circRNAs have been revealed: circRNA can interact with DNA, RNA, or proteins to regulate the biological processes; and the function of circRNA gradually becomes one of the research hot spots [19,20]. Conceptually, circRNA is a highly abundant type of RNA with a long half-life period and therefore becomes a promising biomarker in human diseases [14]. In cancer biology, circRNAs exert their biological functions through multiple mechanisms [20–22]. Among these different mechanisms, the most concerning is the ceRNA mechanism, and this mechanism suggests that circRNA sequences contain miRNA response elements and competitively bind with miRNAs, reduce miRNAs availability, and inhibit the ability of miRNAs to target mRNAs, thus playing an important role in cancer progression [23]. For example, in NSCLC, circ_0001287 represses the proliferation, metastasis, and radiosensitivity of NSCLC cells by decoying miR-21 and inducing the expression of phosphatase and tensin homolog [24]. A previous study of bioinformatics analysis suggests that circ_0002360 was highly expressed in lung adenocarcinoma [12]. However, this study did not validate this finding via detecting its expression in tissue samples, and the biological function of circ_0002360 was still unclear. In this work, we found that circ_0002360 expression was increased in NSCLC tissues, and we validated that circ_0002360 facilitated the proliferative, migrative, and invasive abilities of NSCLC cells with *in vitro* experiments. Our study supports that circ_0002360 is an oncogenic circRNA in the pathogenesis of NSCLC.

MiRNAs can modulate various signaling pathways in tumor cells, via targeting and inhibiting the translation of mRNAs or reducing mRNA stability [25]. For example, miR-449b-3p inhibits epithelial–mesenchymal transition (EMT) of NSCLC cells by targeting IL-6 and inactivating the JAK2/STAT3 signaling pathway [26]. MiR-128-3p regulates metastasis and chemoresistance of NSCLC cell via activating the Wnt/β-catenin and TGF-β pathways [27]. Reportedly, miR‑145‑5p, miR-585-3p, miR-758-3p, and miR-127-5p are lowly expressed in NSCLC [28–31]. In this work, we confirmed that the above microRNAs were the targets of circ_0002360. Our findings suggest that the abnormal overexpression of circ_0002360 is an important upstream mechanism of the dysregulation of these miRNAs. In addition, the above four miRNAs can reverse the promoting effects of circ_0002360 on the malignancy of NSCLC cells. These findings indicate that circ_0002360 can promote the progression of NSCLC depending on miR-127-5p, miR-145-5p, miR-585-3p, and miR-758-3p.

Matrix metalloproteinases (MMPs) belong to a family of zinc-dependent proteases, which are capable of degrading the extracellular matrix (ECM), and MMPs are regarded as vital regulators in modulating ECM remodeling, and they mediate the proliferation, metastasis, and angiogenesis of cancer cells [32–35]. As a crucial member of the MMP family, MMP16 can also display proteolytic activity against components of the ECM, and MMP16 is highly expressed in many types of human cancers and promotes their progression [36]. Reportedly, MMP16 is overexpressed in melanoma, gastric cancer, and glioma, and its high expression implies the adverse prognosis of the patients [36–38]. In NSCLC, it is reported that miR-146-5p can inhibit the progression of NSCLC by regulating MMP16 expression [39]. Here, we discovered that miR-127-5p, miR-145-5p, miR-585-3p, and miR-758-3p could also regulate MMP16. In addition, circ_0002360 acted as a ceRNA to competitively bind to the above miRNAs and thus up-regulate MMP16 expression in NSCLC cells. Our data suggest that circ_0002360, with the ability to decoy multiple upstream miRNAs of MMP16, may be the dominator to maintain the balance of MMP16 in NSCLC cells.

## Conclusion

Circ_0002360, highly expressed in the NSCLC, promotes the expression of MMP16 and the malignancy of NSCLC cells via regulating multiple miRNAs including miR-127-5p, miR-145-5p, miR-585-3p, and miR-758-3p. This is the first study, to our best knowledge, to investigate the biological function and downstream mechanism of circ_0002360 in NSCLC, which helps to better understand the role of ceRNA network in NSCLC progression.

## Data Availability

The data used to support the findings of this study are available from the corresponding author upon request.

## Research Highlights

- Circ_0002360 is identified as a new oncogenic circRNA in NSCLC, which regulates the malignant biological behavior of cancer cells.
- Several downstream miRNAs of circ_0002360 are identified, including miR-127-5p, miR-145-5p, miR-585-3p, and miR-758-3p.
- Circ_0002360 can positively regulate MMP16 expression via repressing these miRNAs.
